# Diagnostic uplift through the implementation of short tandem repeat analysis using exome sequencing

**DOI:** 10.1038/s41431-024-01542-w

**Published:** 2024-02-02

**Authors:** Jihoon G. Yoon, Seungbok Lee, Jaeso Cho, Narae Kim, Sheehyun Kim, Man Jin Kim, Soo Yeon Kim, Jangsup Moon, Jong-Hee Chae

**Affiliations:** 1 Department of Genomic Medicine, Seoul National University Hospital, Seoul, Republic of Korea; 2 Department of Pediatrics, Seoul National University Children’s Hospital, Seoul, Republic of Korea; 3 Department of Neurology, Seoul National University Hospital, Seoul, Republic of Korea

**Keywords:** Disease genetics, Genetics research, Neurological disorders

## Abstract

To date, approximately 50 short tandem repeat (STR) disorders have been identified; yet, clinical laboratories rarely conduct STR analysis on exomes. To assess its diagnostic value, we analyzed STRs in 6099 exomes from 2510 families with mostly suspected neurogenetic disorders. We employed ExpansionHunter and REViewer to detect pathogenic repeat expansions, confirming them using orthogonal methods. Genotype-phenotype correlations led to the diagnosis of thirteen individuals in seven previously undiagnosed families, identifying three autosomal dominant disorders: dentatorubral-pallidoluysian atrophy (*n* = 3), spinocerebellar ataxia type 7 (*n* = 2), and myotonic dystrophy type 1 (*n* = 2), resulting in a diagnostic gain of 0.28% (7/2510). Additionally, we found expanded *ATXN1* alleles (≥39 repeats) with varying patterns of CAT interruptions in twelve individuals, accounting for approximately 0.19% in the Korean population. Our study underscores the importance of integrating STR analysis into exome sequencing pipeline, broadening the application of exome sequencing for STR assessments.

## Introduction

Short tandem repeats (STRs) are repetitive DNA sequences composed of units typically 2–6 base pairs. These sequences exhibit hyper-mutability and high polymorphism, making them potential contributors to diverse phenotypes and disorders [1]. To date, approximately 50 STR disorders have been identified, predominantly in neuromuscular and neuropsychiatric disorders [2–4]. Although long-read sequencing technologies offer advantages for STR investigations, genomic data generation has primarily relied on short-read sequencing due to its cost-effectiveness in clinical settings. Fortunately, the development of various computational tools, such as Expansion Hunter [5], has facilitated the reliable detection of repeat expansions in short-read datasets. Recent studies have demonstrated the feasibilities of STR analysis in large-scale short-read genomes or exomes [6–11]. Therefore, we aimed to explore the diagnostic utilities of STR analysis for identification of pathogenic repeat expansions using exome sequencing.

## Materials and methods

### Study cohorts and sequencing

The study cohorts comprised 6,099 exomes, derived from 2,510 Korean families with rare diseases, who underwent exome sequencing as part of further diagnostic work-ups (Supplementary Table 1).

### Short tandem repeat analysis

Based on the previous reports [7–9], we utilized ExpansionHunter (v5.0) [5] to detect repeat expansions within the target STRs. We selected 21 loci within 20 genes that were sufficiently covered by exomes (Supplementary Fig. 2) [4], and visually inspected candidates exceeding the pathogenic threshold using the Repeat Expansion Viewer (REViewer v0.2.7; Supplementary Fig. 3) [12].

Please refer to the Supplementary information for more detailed materials and methods used.

## Results

### Identified repeat expansions

In our study, we found that the majority (94.0%) had pediatric-onset diseases, and neurodevelopmental disorders constituted the most prevalent primary disease category at 65.6%, with trio sequencing utilized in 67.9% of the cases. Using ExpansionHunter, we targeted 20 genes with adequate locus coverage to detect pathogenic repeat expansions (Supplementary Fig. 2). Our initial analysis yielded 116 potential repeat expansions above recognized pathogenic thresholds (Supplementary Table 2). These candidates were further examined using REViwer, and genotype calls from regions with low coverage, suboptimal mapping quality, or alignment bias towards specific haplotypes were excluded to eliminate false-positives (Supplementary Fig. 3). Consequently, 35 visually suspected repeat expansions were identified, and subsequent validation confirmed 13 repeat expansions. Through genotype-phenotype correlation, these confirmed expansions led to diagnose 13 individuals (7 probands and 6 parents) within 7 families (Table 1, Supplementary Fig. 5): dentatorubral-pallidoluysian atrophy (DRPLA; *n* = 3), spinocerebellar ataxia type 7 (SCA7; *n* = 2), and myotonic dystrophy type 1 (DM1; *n* = 2).

**Table 1:** Clinical findings and detected repeat expansions in families undergoing exome sequencing.

STR disorder (Gene)	Family	Individual	Sex	Age at evaluation	Clinical findings	EH result	Validation method	Validation result
DRPLA (*ATN1*)	F1	Father	M	52	Ataxia with chorea	22/62	fragment analysis	19/64
		Proband	M	14	DD with regression, epilepsy	21/48	fragment analysis	18/89
	F2	Father	M	44	Gait abnormality	23/61	fragment analysis	20/61
		Proband	F	10	DD with regression, epilepsy	21/60	fragment analysis	18/78
	F3	Father	M	51	Mild cognitive decline	25/53	fragment analysis	22/58
		Proband	M	15	DD, epilepsy, microcephaly, ataxia, involuntary movement	19/63	fragment analysis	16/70
SCA7 (*ATXN7*)	F4	Mother	F	55	Cerebellar ataxia with foveal atrophy	10/47	fragment analysis; Nanopore	10/41; 9/42
		Proband	F	29	Cerebellar ataxia with foveal atrophy	10/47	fragment analysis; Nanopore	10/41; 6/47
	F5	Father	M	38	Asymptomatic	10/42	fragment analysis	10/42
		Proband	M	6	DD with regression, diffuse cerebellar atrophy	10/36	fragment analysis	10/92
DM1 (*DMPK*)	F6	Mother	F	35	Myotonia of tongue and grip	13/62	Southern blotting	13/617
		Proband	M	5	DD, hypotonia, elevated CK levels, myopia	43/64	Southern blotting	5/1171
	F7	Father	M	55	Asymptomatic (pre-mutation)	13/44	fragment analysis	NA
		Proband	M	22	Foot deformity, neck webbing, myopathic electromyography	26/51	fragment analysis	26/57

*EH* ExpansionHunter, *M* male, *F* female, *DD* developmental delay, *CK* creatinine kinase, *DRPLA* dentatorubral-pallidoluysian atrophy, *SCA7* spinocerebellar ataxia type 7, *DM1* myotonic dystrophy type 1, *NA* not applicable.

**Fig. 1:**
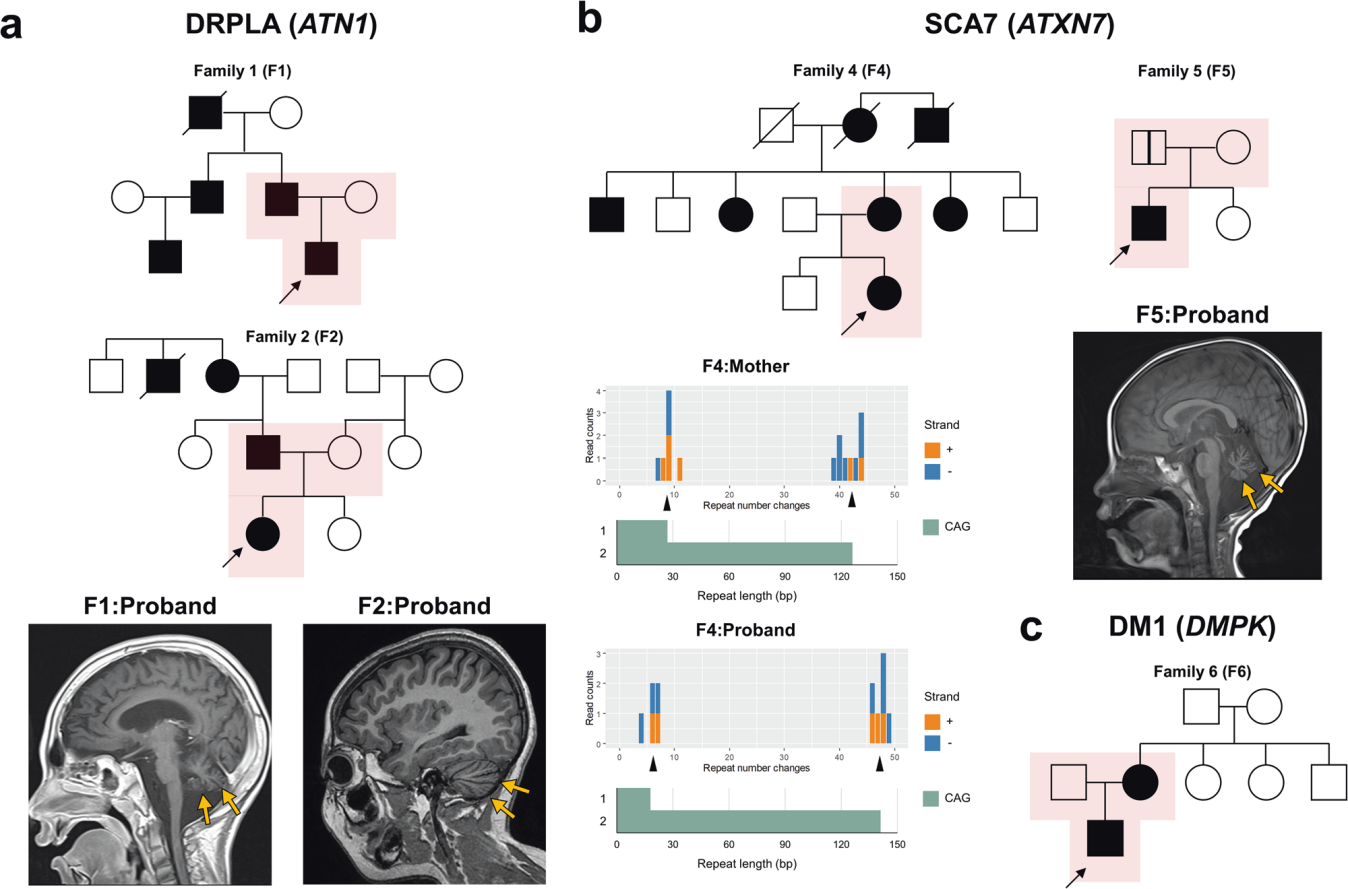
Pedigree and brain imaging findings in representative cases identified by short tandem repeat (STR) analysis. **a** Dentatorubral-pallidoluysian atrophy (DRPLA) was incidentally identified by STR analysis in families 1 and 2. Trio exome sequencing was conducted due to the initial observation of developmental delay in the probands. Initially, both fathers were asymptomatic; however, relevant clinical findings emerged during the follow-up periods. The pedigrees were reconstructed after genotype-phenotype correlation, confirmation of DRPLA, and cascade screening for other affected members. **b** Spinocerebellar ataxia type 7 (SCA7) identified in families 4 and 5. The proband and her mother from family 4 underwent exome sequencing after receiving negative results from SCA panel tests at another institution. However, ExpansionHunter suggested repeat expansions in SCA7. Subsequent fragment analysis and Cas9-mediated Nanopore long-read sequencing cross-validated that the previous results was false negative. Cascade screening of family 4 further confirmed *ATXN7* repeat expansions in other family members. In family 5, *ATXN7* repeat counts estimated by ExpansionHunter were 10/36 in the proband and 10/42 in his father, respectively. Subsequent fragment analysis confirmed the repeat expansions (92 and 42 repeats, respectively). **c** Myotonic dystrophy type 1 (DM1) identified in family 6. Despite ExpansionHunter identifying 64 and 62 repeats in the proband and his mother, respectively, Southern blotting confirmed the presence of extremely long repeats in the proband (1171 repeats) and his mother (617 repeats). During the reverse phenotyping of the mother, myotonia of the tongue and grip were noticed. Brain magnetic resonance imaging revealed cerebellar atrophy in the probands, indicated by yellow arrows. Samples that underwent exome sequencing within the families are highlighted in pink. Black arrows denote the probands within the families.

In the case of DRPLA (families 1–3), the three probands were initially referred to the clinic for developmental delays, and their parents were asymptomatic at the time of initial enrollment. The expanded alleles were found to be transmitted from their fathers, who later developed adult-onset DRPLA symptoms in their 40 s. Brain MRI scans of the probands from families 1 and 2 revealed cerebellar atrophy, and cascade screening within these two families uncovered additional patients with DRPLA who presented with cerebellar ataxia (Fig. 1a).

Repeat expansions in *ATXN7* were detected in two families (families 4 and 5), one with adult-onset symptoms and the other with childhood-onset symptoms in the probands (Fig. 1b). In family 4, the affected members commonly showed signs of cerebellar ataxia and foveal atrophy. These repeat expansions were confirmed using two orthogonal methods (fragment analysis and Nanopore long-read sequencing) following a previously negative result on SCA panels from another institution, which were later determined to be false negatives. After the diagnosis, additional family members were also found to have the repeat expansions. In family 5, the proband and his father had repeat counts estimated at 10/36 and 10/42 by ExpansionHunter, respectively. Fragment analysis later confirmed these expansions to be 92 repeats for the proband and 42 for the father. Although the proband initially exhibited only developmental delays, a regression and cerebellar ataxia were noted at 6 years of age. Particularly, anticipation was evident in this family; the proband was diagnosed with childhood-onset SCA7 before the father with the pathogenic repeat expansion became symptomatic [13].

For DM1, the repeat counts were validated using either fragment analysis or Southern blotting, depending on the length of the repeats. In family 6, ExpansionHunter estimated the repeat count to be 64 repeats in the proband, while Southern blotting revealed an exceptionally long CTG repeat expansion of 1171 repeats, categorized as the congenital type of DM1. This allele was inherited from his mother (617 repeats), who exhibited tongue and grip myotonia during reverse phenotyping (Fig. 1c). In family 7, the proband had a repeat count of 57 CTG repeats, indicative of the mild type of DM1. He exhibited mild muscle weakness with skeletal anomalies, including foot deformities and neck webbing. Recent electromyography revealed myopathic findings. The expanded allele was inherited from his father, who had 44 CTG repeats, falling within the premutable range (35–49 repeats).

### Expanded *ATXN1* alleles with CAT interruptions

Among the 35 repeat expansions initially suspected through visual inspection, 22 were not subjected to further confirmatory methods, as subsequent evaluation deemed them likely non-pathogenic. Within the *AR* gene, we identified ten heterozygous repeat expansions (≥38 repeats) in females (5 alleles with 38 repeats, 2 alleles with 39 repeats, 2 alleles with 40 repeats, and 1 allele with 41 repeats). The female carrier frequency of the expanded allele was 0.52% (9 unrelated alleles in 1,746 mothers within trio- or quartet-sequenced samples). Additionally, we identified twelve expanded *ATXN1* alleles (≥39 repeats) with interruptions, none of which were associated with clinical features of SCA1 at the time of evaluation. We observed that the presence of different thymidine (T) nucleotides within the interruptions allowed for accurate phasing and alignment (Supplementary Fig. 6). We found six different patterns of interruptions, where each expanded allele had either two or four CAT interruptions, leading to amino acid changes from glutamate (Q) to histidine (H) residues. Notably, the (Q)_26-31_(H)(Q)(H)(Q)_10_ motifs were the predominant pattern observed in 9 individuals, as previously reported [14]. After excluding four related alleles, we identified eight expanded/interrupted alleles among 8512 alleles originating from 4256 unrelated individuals. This suggests that expanded/interrupted *ATXN1* alleles may be present in approximately 0.19% of the Korean population (Supplementary Table 3).

## Discussion

Our approach utilized ExpansionHunter and REViewer for screening repeat expansions and visually inspecting aligned reads, respectively, and we validated them using orthogonal methods. After these processes and genotype-phenotype correlations, we identified thirteen individuals from seven previously undiagnosed families across three distinct disorders. Our cohort primarily consisted of pediatric patients with neurodevelopmental or neuromuscular disorders, with repeat expansions confirmed in the *ATN1*, *ATXN7*, and *DMPK* genes. The overall diagnostic gain (0.28%, 7/2,510) was comparable to a previous study of a movement disorder cohort (0.24%, 7/2,867) [8]. This study involved six genes (*ATXN1*, *ATXN3*, *ATXN7*, *HTT*, *NOP56*, and *PPP2R2B*), while a higher detection rate has been reported in a spinocerebellar ataxia cohort (4.4%, 22/498) [10], which included five genes (*ATXN2*, *ATXN3*, *NOP56*, *AR* and *HTT*). Also, we incidentally found different patterns of expanded *ATXN1* alleles with interruptions in twelve individuals who did not report SCA1-related phenotypes. These findings highlight the applications of STR analysis, which is often overlooked in exome analysis.

The detection capacity for repeat expansions using exomes strongly relies on read length and locus coverage [6]. The discrepancies observed between ExpansionHunter estimates and results from orthogonal methods emphasize the challenges in accurately estimating repeat counts with short-reads (Table 1), which can be significantly influenced by the number of reads anchored into the targeted regions. Particularly, we could not assess the *FMR1* region located on the X chromosome due to insufficient coverage (Supplementary Fig. 2b), despite it being one of the most common causes of repeat expansion diseases in the pediatric population. Moreover, our study may underrepresent the actual frequency of repeat expansions, as false-negative results are possible in outliers with low coverage (Supplementary Fig. 4) [9]. Consequently, repeat counts estimated by ExpansionHunter require cautious interpretation and should be confirmed with orthogonal methods for accurate repeat count assessment.

Internal sequence interruptions have been implicated in disease phenotypes, penetrance, and age of onset of various STR disorders [4]. We found interruptions within expanded *ATXN1* alleles and observed intriguing patterns. These interruptions in the polyQ tract are understood to mitigate aggregate formation and increase the stability of repeat transmission to offspring, which may contribute to the absence of symptoms or a delayed onset age seen in SCA1 [15]. A previous study reported expanded/uninterrupted alleles in 1.40% of Korean patients with cerebellar ataxia, and their onset age ranged from 44 to 59 years [16]. Therefore, it remains uncertain whether the carriers in our study might develop SCA1-related symptoms later in life. However, the proportions of expanded/interrupted alleles appeared to be much lower (0.19%) than the previous study, with the (Q)_26-31_(H)(Q)(H)(Q)_10_ motifs being revealed as the most common patterns in the Korean population.

In conclusion, this study, which encompassed a substantial number of pediatric patients and samples sequenced as trios or quartets within the East Asian population, serves to broaden the molecular spectrum and enhance the applicability of exome sequencing for STR assessments. The integration of STR analysis into the exome sequencing pipeline holds the potential to provide additional diagnostic opportunities.

### Supplementary information

Supporting Information

## Data Availability

Data and materials are available upon reasonable request.

## Code availability

WARP, https://github.com/broadinstitute/warp. STR catalogs, https://github.com/Illumina/RepeatCatalogs. Expansion Hunter, https://github.com/Illumina/ExpansionHunter. Repeat Expansion Viewer, https://github.com/Illumina/REViewer.

## References

[1] Depienne C, Mandel JL (2021). 30 years of repeat expansion disorders: What have we learned and what are the remaining challenges?. Am J Hum Genet.

[2] Trost B, Engchuan W, Nguyen CM, Thiruvahindrapuram B, Dolzhenko E, Backstrom I (2020). Genome-wide detection of tandem DNA repeats that are expanded in autism. Nature.

[3] Mojarad BA, Engchuan W, Trost B, Backstrom I, Yin Y, Thiruvahindrapuram B (2022). Genome-wide tandem repeat expansions contribute to schizophrenia risk. Mol Psychiatry.

[4] Chintalaphani SR, Pineda SS, Deveson IW, Kumar KR (2021). An update on the neurological short tandem repeat expansion disorders and the emergence of long-read sequencing diagnostics. Acta Neuropathol Commun.

[5] Dolzhenko E, Deshpande V, Schlesinger F, Krusche P, Petrovski R, Chen S (2019). ExpansionHunter: a sequence-graph-based tool to analyze variation in short tandem repeat regions. Bioinformatics.

[6] Tang H, Kirkness EF, Lippert C, Biggs WH, Fabani M, Guzman E (2017). Profiling of Short-Tandem-Repeat Disease Alleles in 12,632 Human Whole Genomes. Am J Hum Genet.

[7] Tankard RM, Bennett MF, Degorski P, Delatycki MB, Lockhart PJ, Bahlo M (2018). Detecting Expansions of Tandem Repeats in Cohorts Sequenced with Short-Read Sequencing Data. Am J Hum Genet.

[8] van der Sanden BPGH, Corominas J, de Groot M, Pennings M, Meijer RPP, Verbeek N (2021). Systematic analysis of short tandem repeats in 38,095 exomes provides an additional diagnostic yield. Genet Med.

[9] Ibañez K, Polke J, Hagelstrom RT, Dolzhenko E, Pasko D, Thomas ERA (2022). Whole genome sequencing for the diagnosis of neurological repeat expansion disorders in the UK: a retrospective diagnostic accuracy and prospective clinical validation study. Lancet Neurol.

[10] Méreaux JL, Davoine CS, Coutelier M, Guillot-Noël L, Castrioto A, Charles P (2023). Fast and reliable detection of repeat expansions in spinocerebellar ataxia using exomes. J Med Genet.

[11] Shi Y, Niu Y, Zhang P, Luo H, Liu S, Zhang S (2023). Characterization of genome-wide STR variation in 6487 human genomes. Nat Commun.

[12] Dolzhenko E, Weisburd B, Ibañez K, Rajan-Babu IS, Anyansi C, Bennett MF (2022). REViewer: haplotype-resolved visualization of read alignments in and around tandem repeats. Genome Med.

[13] Bah MG, Rodriguez D, Cazeneuve C, Mochel F, Devos D, Suppiej A (2020). Deciphering the natural history of SCA7 in children. Eur J Neurol.

[14] Chung MY, Ranum LPW, Duvick LA, Servadio A, Zoghbi HY, Orr HT (1993). Evidence for a mechanism predisposing to intergenerational CAG repeat instability in spinocerebellar ataxia type I. Nat Genet.

[15] Menon RP, Nethisinghe S, Faggiano S, Vannocci T, Rezaei H, Pemble S (2013). The role of interruptions in polyQ in the pathology of SCA1. PLoS Genet.

[16] Jang JH, Yoon SJ, Kim SK, Cho JW, Kim JW (2022). Detection Methods and Status of CAT Interruption of ATXN1 in Korean Patients With Spinocerebellar Ataxia Type 1. Ann Lab Med.

