# Supplementary material for: Diagnostic uplift through the implementation of short tandem repeat analysis using exome sequencing
Source: Eur J Hum Genet. 2024 Feb 2;32(5):584–7. doi: 10.1038/s41431-024-01542-w (PMC11061289; doi:10.1038/s41431-024-01542-w)
Supplement: Supplementary file 1 — Supporting Information [file 41431_2024_1542_MOESM1_ESM.pdf]

# Supplementary Information

# Diagnostic uplift through the implementation of short tandem repeat analysis using exome sequencing

Jihoon G. Yoon[1†], Seungbok Lee[1,2†], Jaeso Cho[1,2†], Narae Kim[3], Sheehyun Kim[1], Man Jin Kim[1,4], Soo Yeon Kim[1,2], Jangsup Moon[1,3]* and Jong-Hee Chae[1,2]*

**< Table of Contents >**

**Supplementary Materials and Methods…....................................................2**

**Supplementary References……………….................................................6**

**Supplementary Figures…………………………………………………………….7**

**Fig. S1**. Principal component analysis (PCA) for ancestry inference …………..………..7
**Fig. S2**. Target gene selection process for analysis ………..……………………..…8
**Fig. S3**. Study workflow and visual inspection artifacts ……….……………………9
**Fig. S4**. Distribution of estimated repeat counts across target genes ………..…………10
**Fig. S5**. REViewer visualization of confirmed expanded alleles ……….…………......11
**Fig. S6**. Patterns of expanded *ATXN1* alleles with CAT interruptions …………........18

**Supplementary Tables…………………………………………………………......21**

**Table S1**. Overview of the exome-sequenced cohort ………...................................21
**Table S2**. Targeted regions for short-tandem repeat analysis ………………………….22
**Table S3**. Frequencies of *ATXN1* expanded alleles with CAT interruptions ..………...23

# Supplementary Materials and Methods

## Study cohorts and sequencing

The study cohorts comprised 6,099 exomes, derived from 2,510 Korean families with rare diseases. These families had undergone exome sequencing as part of further diagnostic work-ups following negative results on routine molecular tests, such as chromosomal microarray or targeted sequencing. Solved cases in short tandem repeat (STR) disorders were not included, and prior PCR tests for repeat expansions were either not conducted or yielded negative results. The probands were mostly suspected of having neurogenetic disorders. In pediatric patients, these conditions included neurodevelopmental disorders, neuromuscular diseases, or other rare diseases, while adult patients included cerebellar ataxia, hereditary spastic paraplegia, or other rare disease patients (**Supplementary Table 1**). These participants were recruited from the rare disease centers of two hospitals, namely Seoul National University Hospital and Seoul National University Bundang Hospital, Seoul and Seongnam, Republic of Korea, respectively. Informed consent was duly obtained from all participants, and the study was granted approval by the internal review board of Seoul National University Hospital (IRB No. 1406-081-588, 2006-083-1132).

## Exome sequencing and data processing

Whole blood was obtained from the probands and their parents. Genomic DNA was extracted using the QIAamp DNA Blood Mini Kit (Qiagen, Hilden, Germany) following the

manufacturer's protocol. A SureSelect XT Human All Exon V5/V6 Kit (Agilent Technologies Inc., CA, USA) was used for hybridization. Genomic DNA samples were sequenced using a NovaSeq 6000 system (Illumina, CA, USA). Sequenced reads of 150 base pairs in lengths were aligned to the human reference genome hg38, which includes decoys, sourced from the Genomics Public Data on Google Cloud (https://console.cloud.google.com/storage/browser/genomics-public-data/resources/broad/hg38/v0/).

We utilized the WDL Analysis Research Pipelines (WARP; Broad Institute, MA, USA) for robust and reproducible analysis, employing the Exome Germline Single Sample pipeline (v3.0.4). The alignment process was performed within the WARP pipeline using the Burrow-Wheeler Aligner mem (0.7.15) with alt-aware manner. Genetic ancestry inference with principal component analysis was conducted using Peddy (v0.4.8; **Supplementary Fig. 1**)[1].

**Short tandem repeat analysis**

Based on the previous studies[2–4], we employed ExpansionHunter (v5.0)[5] to detect the repeat expansions within the target STRs. From 47 candidate regions identified in the literature[6], we focused on 21 loci within 20 genes that demonstrated a median locus coverage (LC) value greater than 20 across our samples (**Supplementary Fig. S2**, **Supplementary Table S2**). ExpansionHunter was run with the configurations below: --sex: "male or female" {sex information of the samples}, --min-locus-coverage "20", --analysis-mode "seeking". We established a minimum LC threshold (--min-locus-coverage) of 20, as our analysis indicated that samples with LC values below this threshold

consistently led to false calls and misalignments upon visual inspection. Genotypes that met these criteria and were identified by ExpansionHunter were automatically labeled as 'PASS.' These genotypes were then subjected to visual inspection using Repeat Expansion Viewer (REViewer v0.2.7)[7] to rule out potential false positives exceeding the pathogenic threshold. Genotype calls with low read coverage (less than 5x) in REViewer, poor read alignment quality, or haplotype-specific alignment bias were discarded from further analysis (**Supplementary Fig. 3**). We used the software *R* and 'ggplot2' package to visualize our data[8].

## Confirmation of repeat expansions

After excluding false calls by visual inspection and genotype-phenotype correlation assessment, we validated 13 repeat expansions (**Supplementary Fig. S5**) using several orthogonal methods, including fragment analysis, Southern blot, or Nanopore long-read sequencing. For the confirmation of expanded alleles in dentatorubral-pallidoluysian atrophy (DRPLA), myotonic dystrophy type 1 (DM1), and spinocerebellar ataxia type 7 (SCA7) patients, fragment analysis was employed. Southern blot hybridization was performed using the pM10M-6 probe to detect long expansions (>1,000 repeats) in the *DMPK* gene. Furthermore, expanded alleles in a SCA7 family were confirmed using nanopore long-read sequencing[9].

## Cas9-mediated Nanopore sequencing

To perform Cas9-mediated Nanopore sequencing, genomic DNA was extracted from whole blood samples using the Qiagen Puregene blood kit (Qiagen, Maryland, MD, USA; cat. 158023). Cas9-mediated target enrichment for the *ATXN7* gene was carried out as previously described[10] with some modifications, utilizing the following gRNAs:

ATXN7-gRNA1: 5'-AAAAATTGAAAATCTGCATA-3';

ATXN7-gRNA2: 5'-TTAATTTTTTAAGCCCAGGC-3'.

In this study, a total of 5 μg of DNA was utilized, following the Cas9 sequencing kit protocol (cat. SQK-CS9109; Oxford Nanopore Technologies, UK). The prepared libraries were loaded onto R9.4 flow cells (FLO-MIN107) and sequenced using the GridION platform from Oxford Nanopore Technology. Base calling and FASTQ conversion were performed using the MinKNOW (v5.3.6). The resulting FASTQ files were aligned to the human reference genome hg38 using minimap2 (v2.24-r1122). To estimate the CAG repeat counts in the *ATXN7* gene, the software Straglr (v1.4.1)[11] was employed.

# Supplementary References


1. Pedersen BS, Quinlan AR. Who's Who? Detecting and Resolving Sample Anomalies in Human DNA Sequencing Studies with Peddy. Am J Hum Genet. 2017;100(3):406–13.

2. Tankard RM, Bennett MF, Degorski P, Delatycki MB, Lockhart PJ, Bahlo M. Detecting Expansions of Tandem Repeats in Cohorts Sequenced with Short-Read Sequencing Data. Am J Hum Genet. 2018;103(6):858–73.

3. van der Sanden BPGH, Corominas J, de Groot M, Pennings M, Meijer RPP, Verbeek N, et al. Systematic analysis of short tandem repeats in 38,095 exomes provides an additional diagnostic yield. Genet Med. 2021;23(8):1569–73.

4. Ibañez K, Polke J, Hagelstrom RT, Dolzhenko E, Pasko D, Thomas ERA, et al. Whole genome sequencing for the diagnosis of neurological repeat expansion disorders in the UK: a retrospective diagnostic accuracy and prospective clinical validation study. Lancet Neurol. 2022;21(3):234–45.

5. Dolzhenko E, Deshpande V, Schlesinger F, Krusche P, Petrovski R, Chen S, et al. ExpansionHunter: a sequence-graph-based tool to analyze variation in short tandem repeat regions. Bioinformatics. 2019 Nov 1;35(22):4754–6.

6. Chintalaphani SR, Pineda SS, Deveson IW, Kumar KR. An update on the neurological short tandem repeat expansion disorders and the emergence of long-read sequencing diagnostics. Acta Neuropathol Commun. 2021;9(1):98.

7. Dolzhenko E, Weisburd B, Ibañez K, Rajan-Babu IS, Anyansi C, Bennett MF, et al. REViewer: haplotype-resolved visualization of read alignments in and around tandem repeats. Genome Med. 2022;14(1):84.

8. Wickham H. ggplot2: Elegant Graphics for Data Analysis [Internet]. New York, NY: Springer; 2009. Available from: https://link.springer.com/10.1007/978-0-387-98141-3

9. Miyatake S, Koshimizu E, Fujita A, Doi H, Okubo M, Wada T, et al. Rapid and comprehensive diagnostic method for repeat expansion diseases using nanopore sequencing. npj Genom Med. 2022;7(1):1–15.

10. Gilpatrick T, Lee I, Graham JE, Raimondeau E, Bowen R, Heron A, et al. Targeted nanopore sequencing with Cas9-guided adapter ligation. Nat Biotechnol. 2020;38(4):433–8.

11. Chiu R, Rajan-Babu IS, Friedman JM, Birol I. Straglr: discovering and genotyping tandem repeat expansions using whole genome long-read sequences. Genome Biol. 2021;22(1):224.

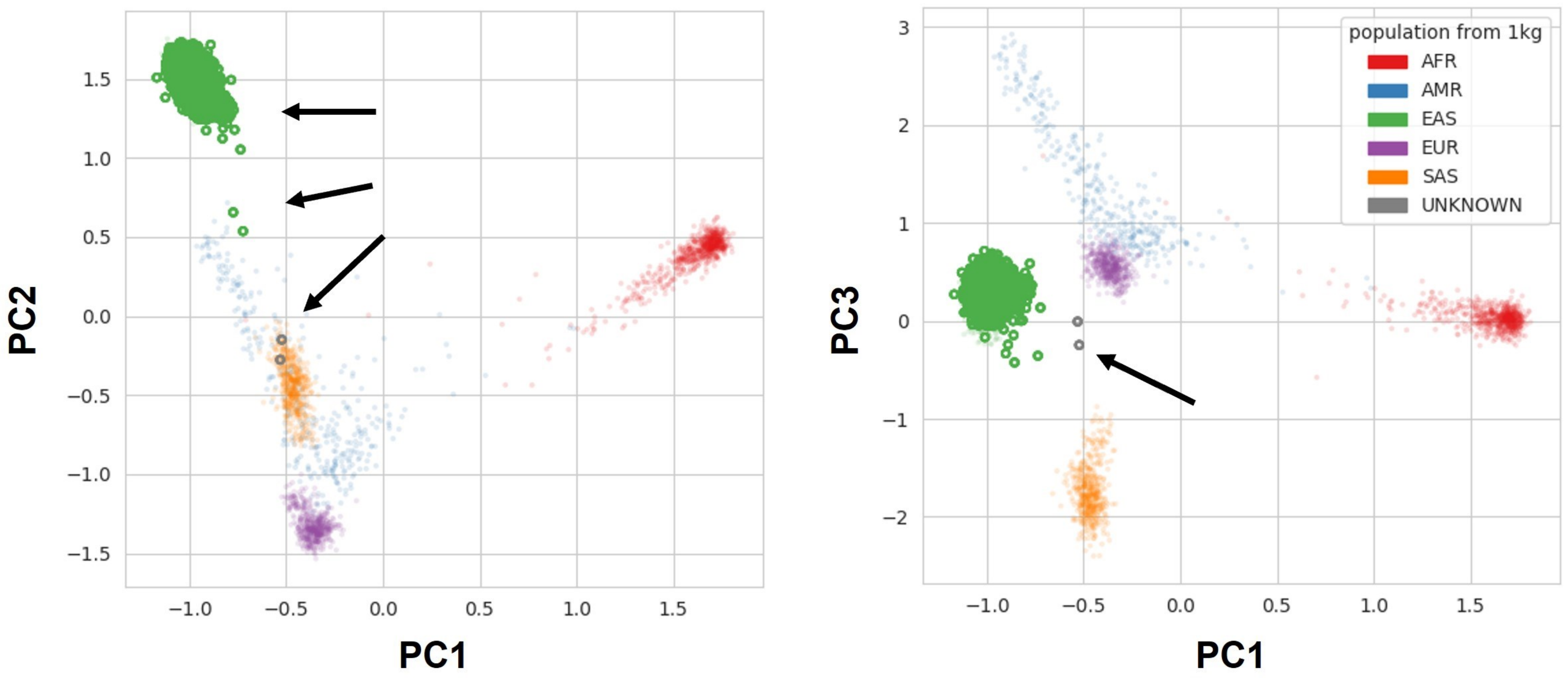


**Supplementary Fig. 1. Principal component analysis (PCA) for ancestry inference.**

PCA of 6,099 exomes, which was enrolled in this study, was performed using Peddy to infer genetic ancestry, with reference populations from the 1000 Genomes Project. The analysis predominantly identifies East Asian (EAS) ancestry, consistent with a Korean cohort (indicated by bold circles; black arrows). Deviations from the main EAS cluster, specifically two between the EAS and South Asian (SAS) clusters and two in the SAS cluster, correspond to probands and their mothers, respectively, who had international marriages, as seen in the trio-sequenced samples.

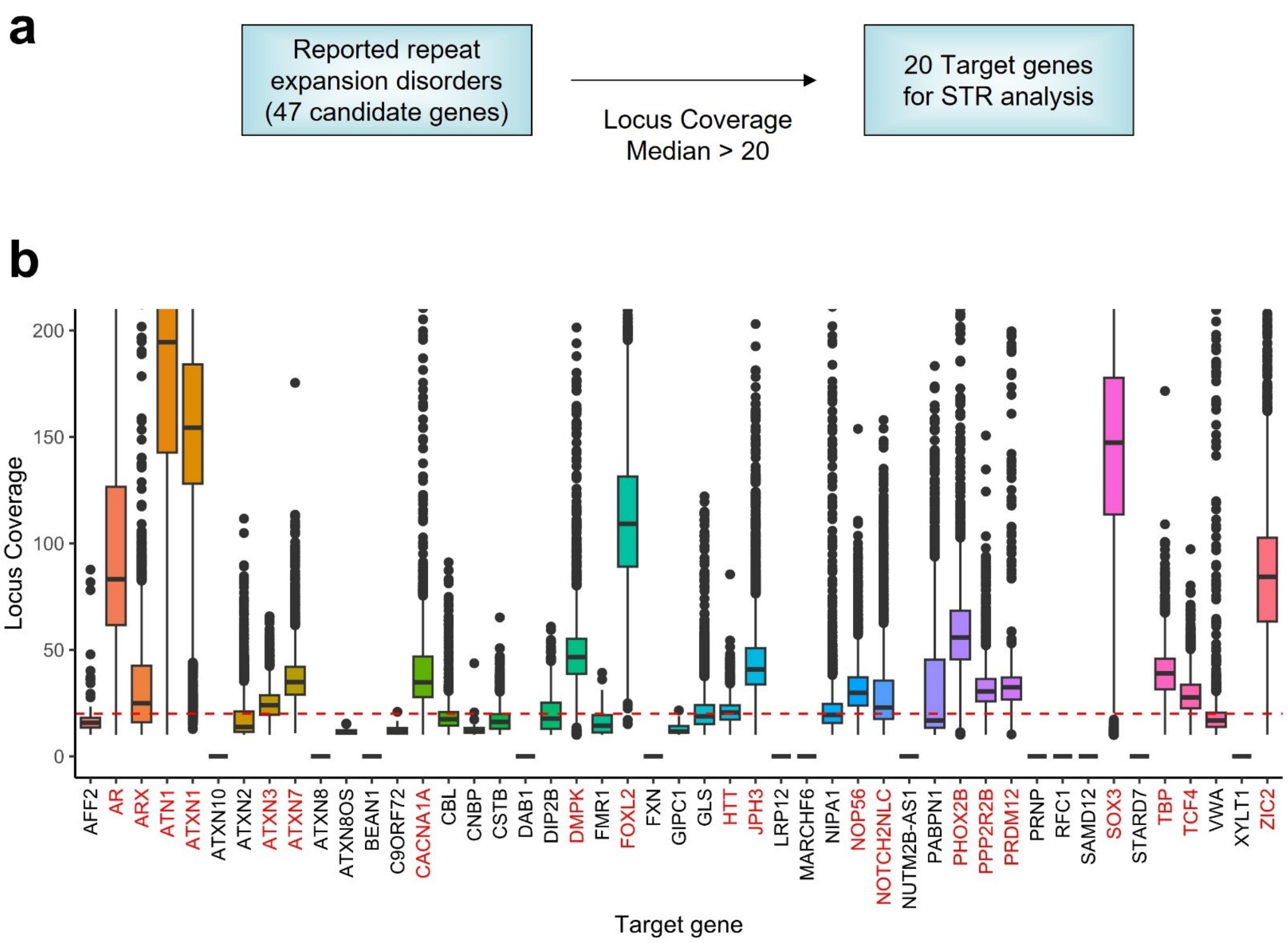


**Supplementary Fig. 2. Target gene selection process for analysis.**

**a**. Schematic representation of the process adopted for target gene selection. Based on literature reviews[6], 47 candidate regions were initially evaluated. Upon visual inspection using REViewer, it was evident that samples with a locus coverage (LC) below 20 almost invariably resulted in false calls for repeat counts. Therefore, we narrowed down our selection to 20 genes, each having a median LC value greater than 20. **b**. Distribution of LC values across the 47 candidate genes. Each box plot represents the spread of locus coverage for a specific region. The red dashed line indicates the threshold of LC = 20. Selected genes are highlighted in red, having a median LC value > 20.

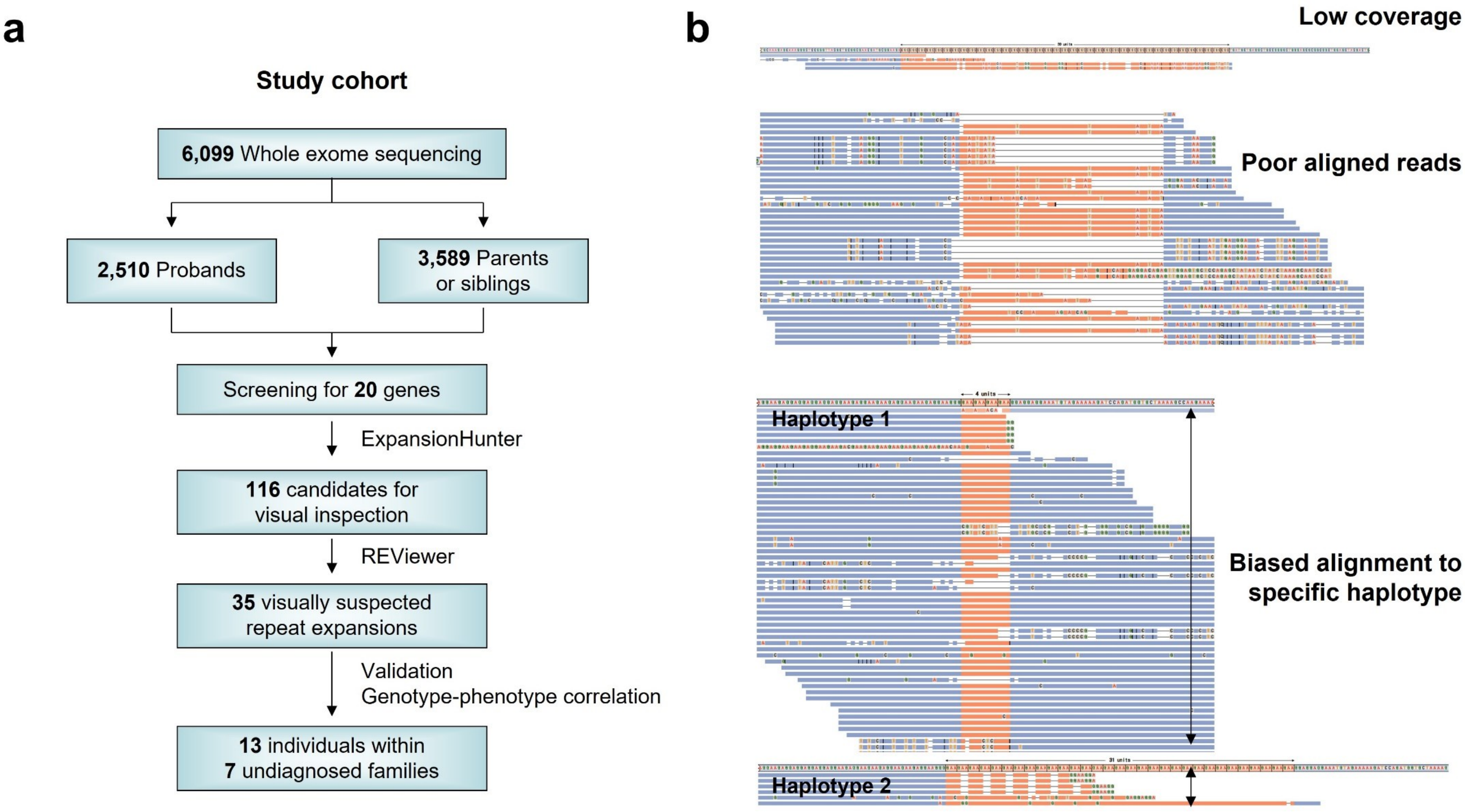


**Supplementary Fig. 3. Study workflow and visual inspection artifacts.**

**a**. Workflow from whole exome sequencing of 6,099 individuals, detailing the steps from gene screening to the identification of undiagnosed cases. **b**. Examples of artifacts that lead to false positive calls during visual inspection, including low coverage, poor read alignment, and haplotype-specific bias. Expanded repeats with these findings were manually excluded and considered as false positive calls.

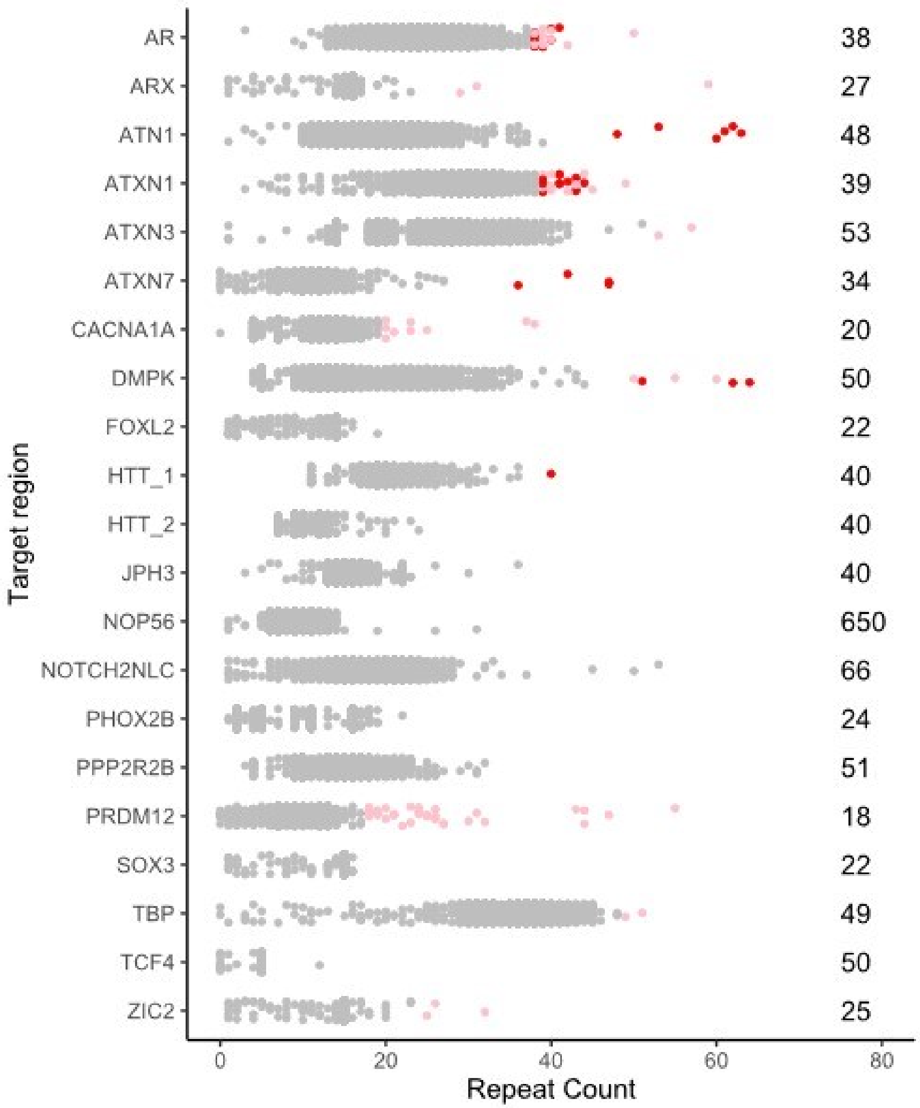


**Supplementary Fig. 4. Distribution of estimated repeat counts across target genes.**

Swim lane plot showing the distribution of repeat counts for each gene across 6,099 exomes, with pathogenic thresholds annotated. Repeat counts above these thresholds are highlighted. Pink dots represent suspected false positives, and red dots indicate potential repeat expansions after visual inspection phase.

**Supplementary Fig. 5. REViewer visualization of confirmed expanded alleles.**

**a-c**. Visualization of expanded *ATN1* alleles found in families 1 through 3 (F1–F3) using REViewer. **d-e**. Visualization of expanded *ATXN7* alleles found in families 4 and 5 (F4, F5) using REViewer. **f-g**. Visualization of expanded *DMPK* alleles found in families 6 and 7 (F6, F7) using REViewer. The figures are presented sequentially by family number. Non-expanded alleles are not depicted due to space constraints.

**a**

**F1:Father (62 units)**

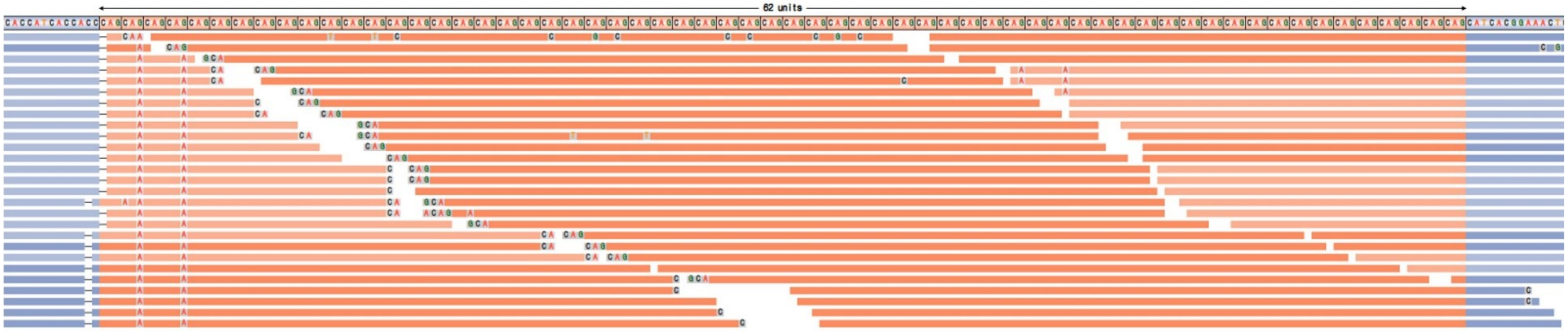


**F1:Proband (48 units)**

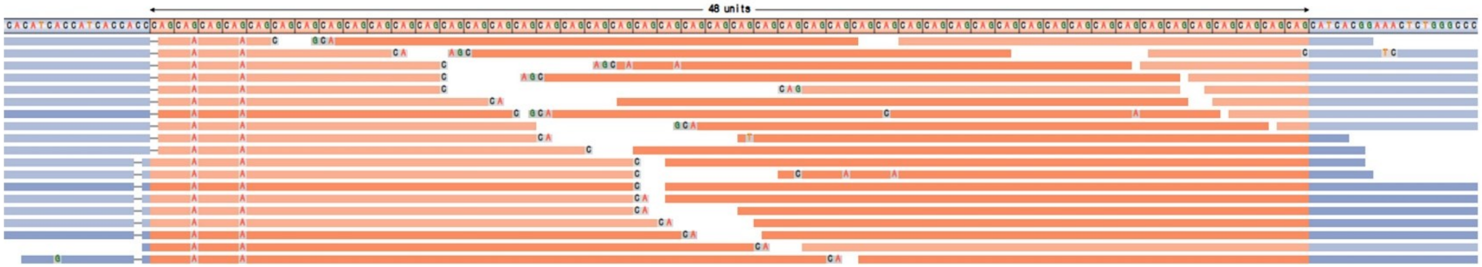

**b**

**F2:Father (61 units)**

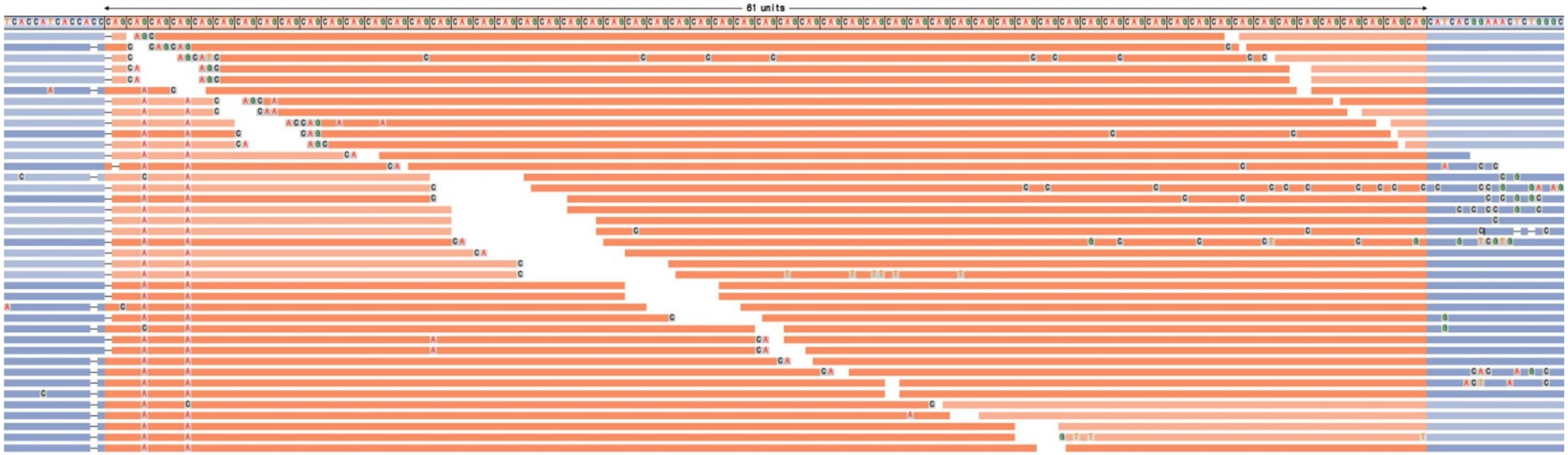


**F2:Proband (60 units)**

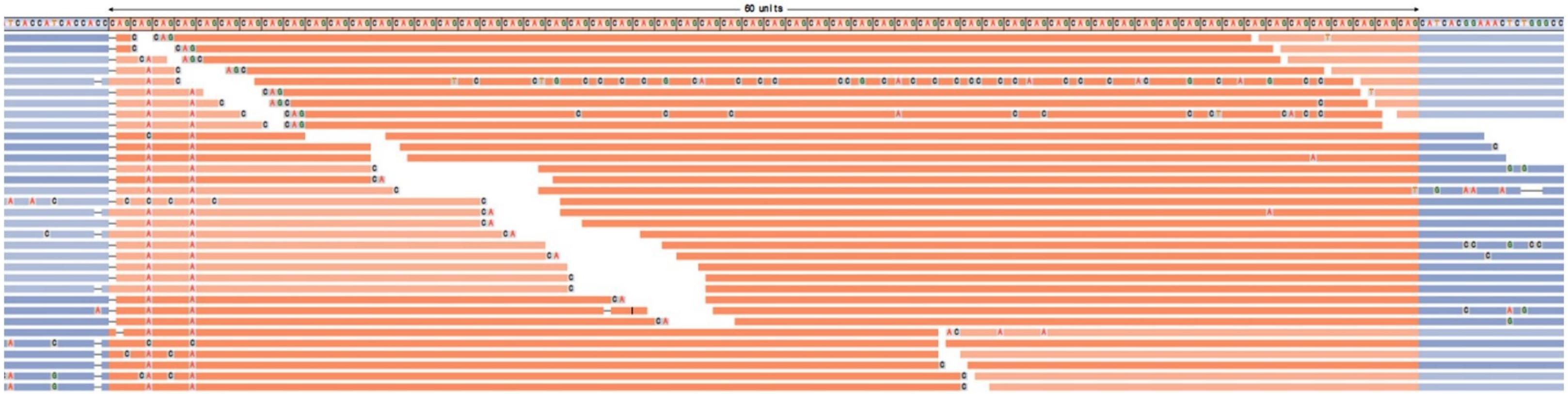

**c**

**F3:Father (53 units)**

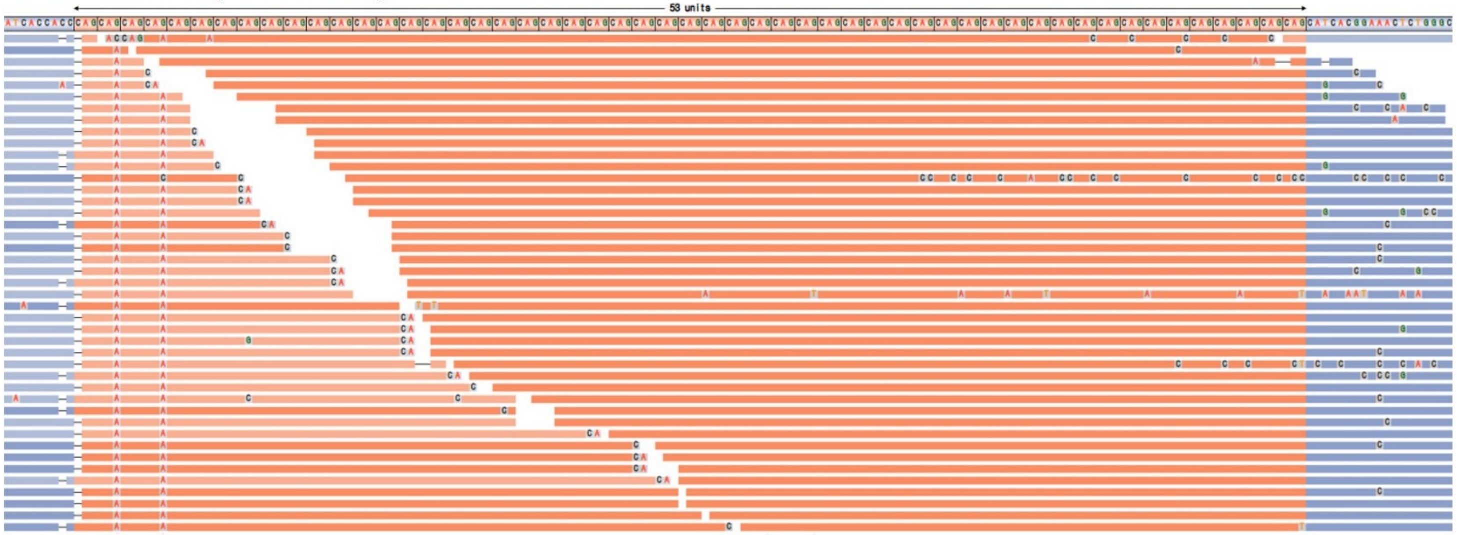

**F3:Proband (63 units)**

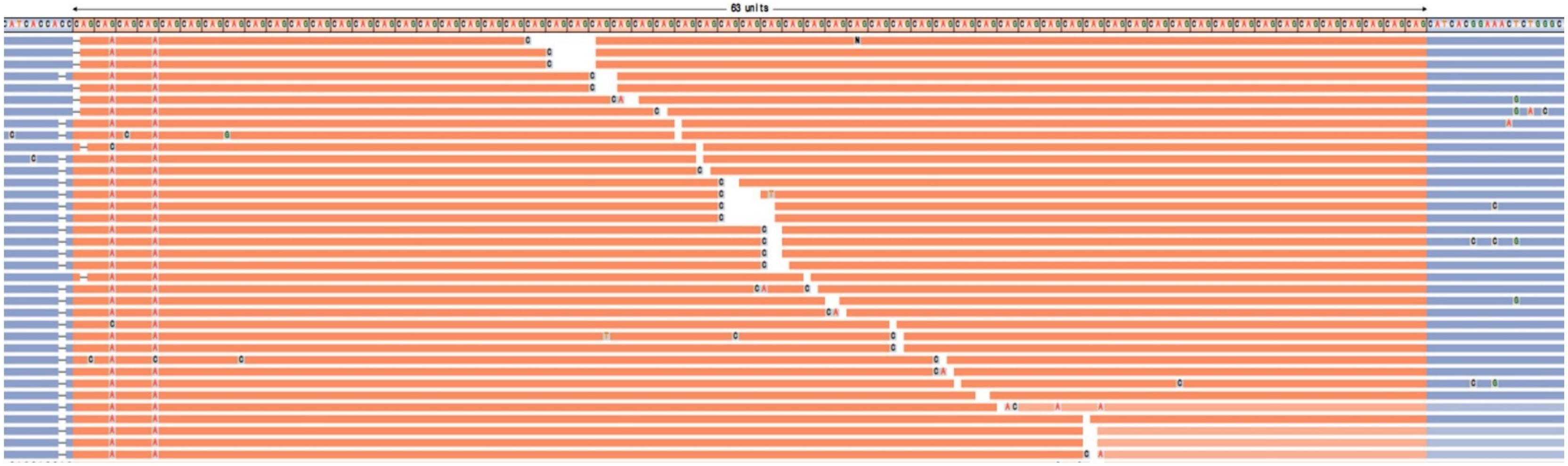

**d**

**F4:Mother (47 units)**

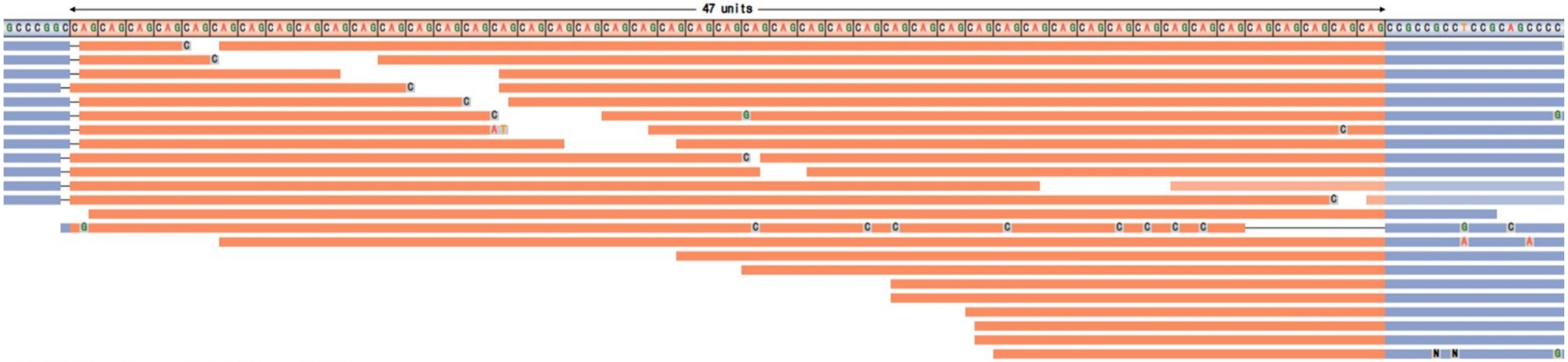


**F4:Proband (47 units)**

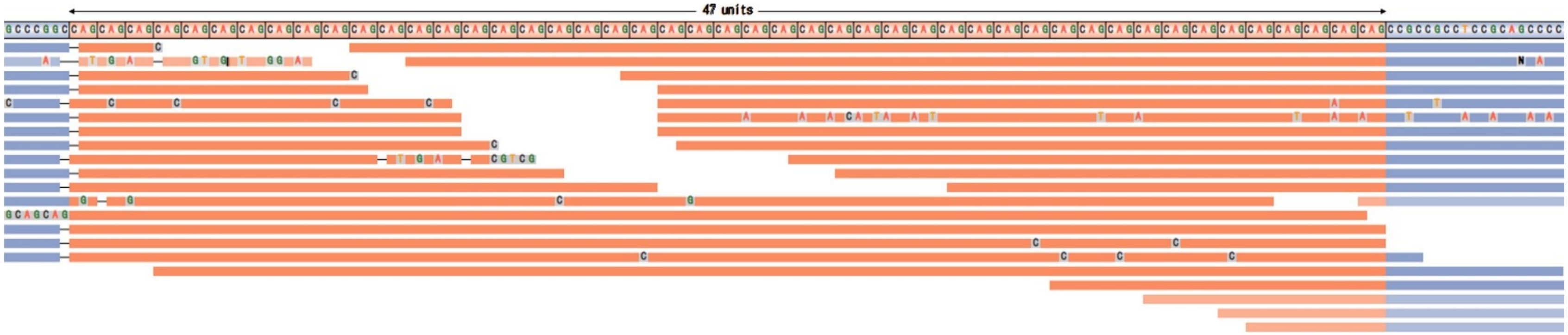

**e**

## F5:Father (42 units)

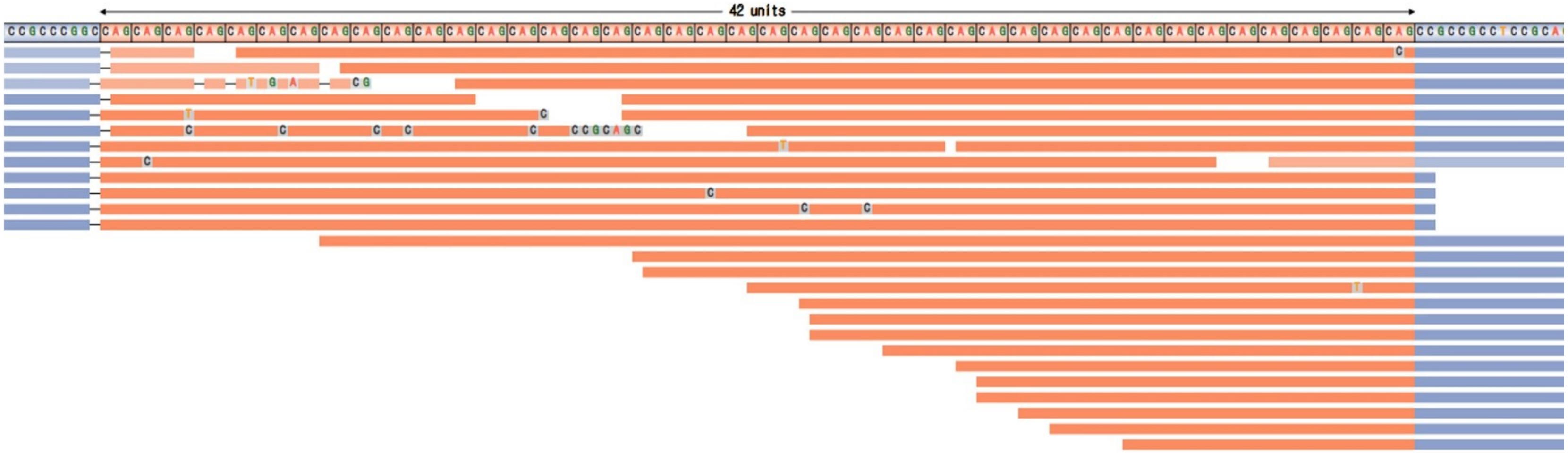


## F5:Proband (36 units)

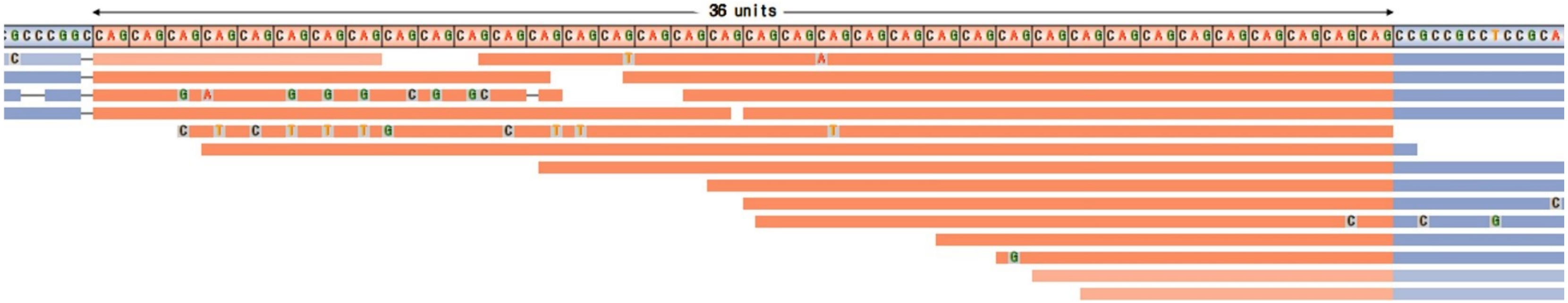

**f**

**F6:Mother (62 units)**

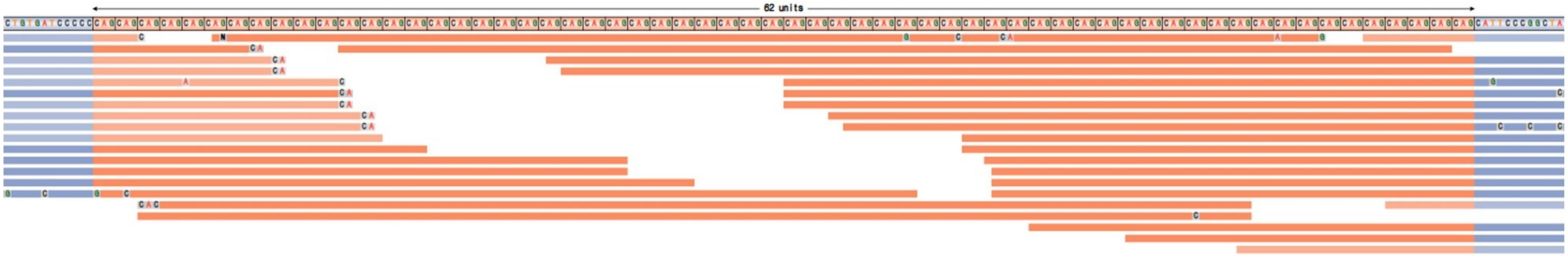


**F6:Proband (64 units)**

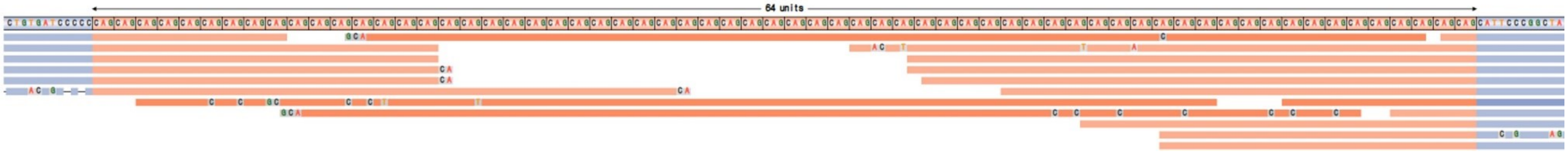

**g**

**F7:Father (44 units)**

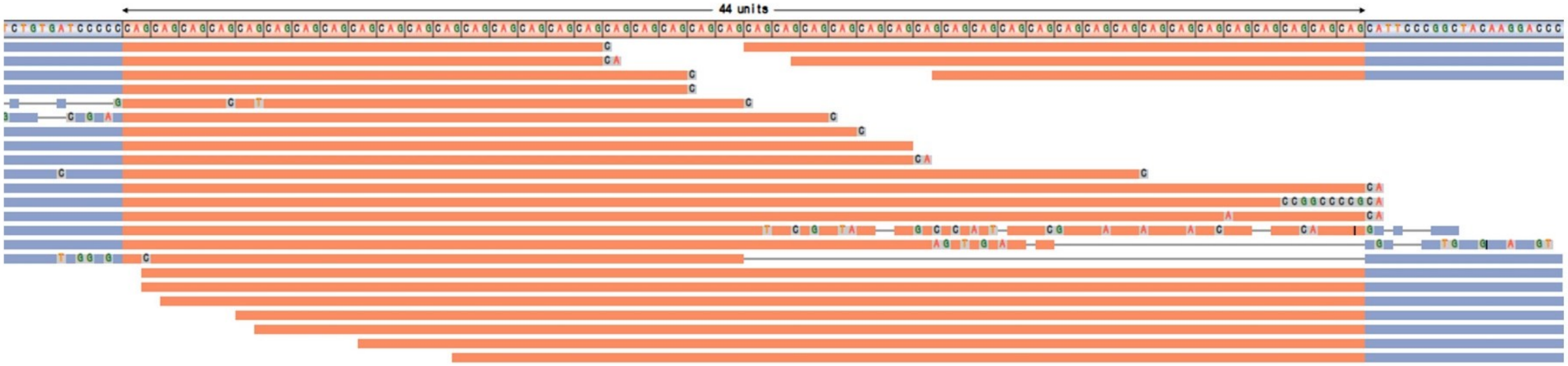


**F7:Proband (51 units)**

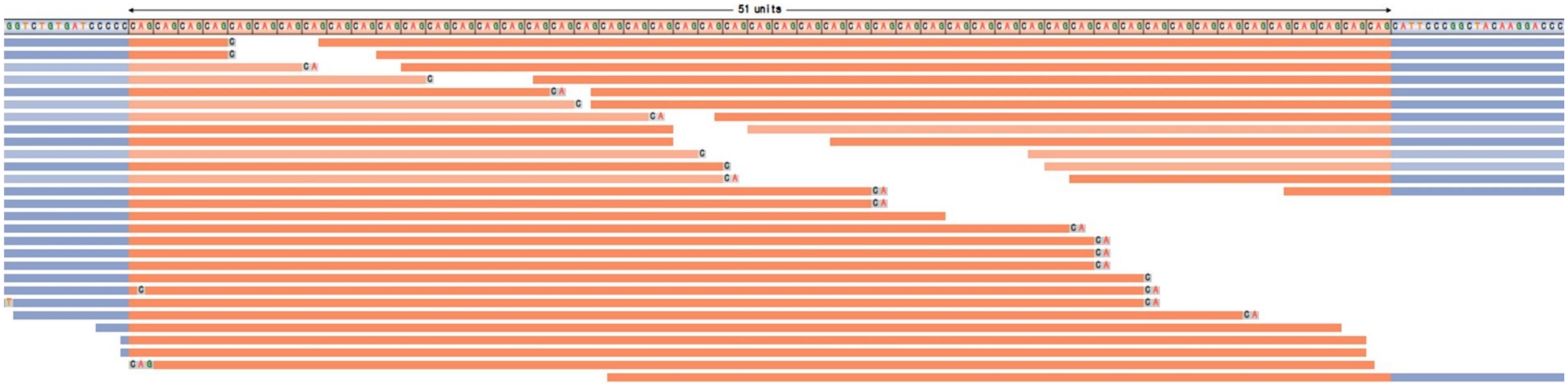

**Supplementary Fig. 6. Patterns of expanded *ATXN1* alleles with CAT interruptions**

**a-f**. Six representative patterns of expanded *ATXN1* alleles (39–44 repeats) with CAT interruptions found in 12 individuals. Refer to Supplementary Table 3 for more detailed information. **a**. 44 repeats with (Q)$_{31}$(H)(Q)(H)(Q)$_{10}$ motif, **b**. 43 repeats with (Q)$_{30}$(H)(Q)(H)(Q)$_{10}$ motif, **c**. 41 repeats with (Q)$_{28}$(H)(Q)(H)(Q)$_{10}$ motif, **d**. 41 repeats with (Q)$_{13}$(H)(Q)(H)(Q)$_{7}$(H)(Q)(H)(Q)$_{15}$ motif, **e**. 39 repeats with (Q)$_{26}$(H)(Q)(H)(Q)$_{10}$ motif, **f**. 39 repeats with (Q)$_{12}$(H)(Q)(H)(Q)$_{9}$(H)(Q)(H)(Q)$_{16}$ motif. Sites of CAT interruptions are highlighted by red rectangles.

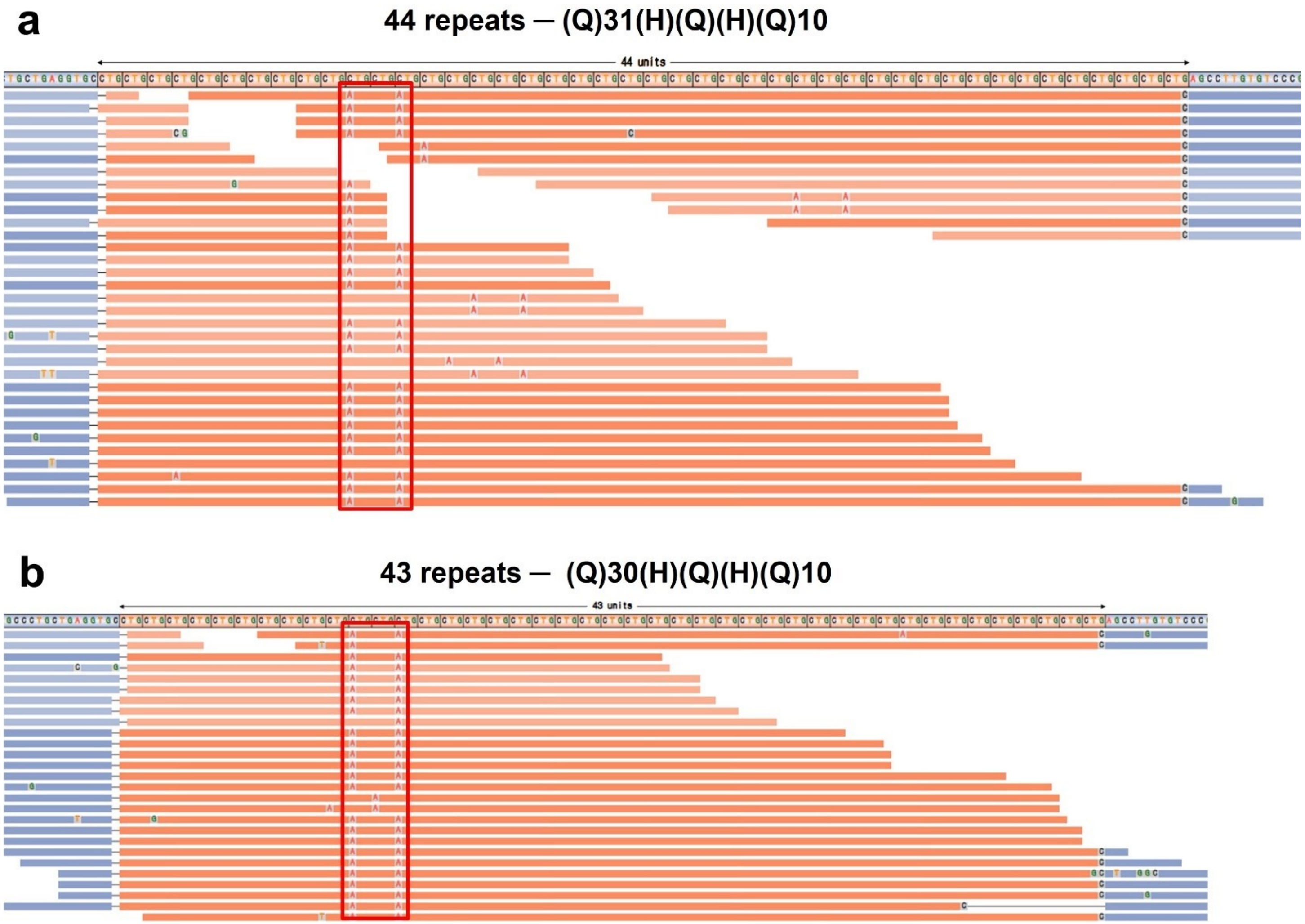

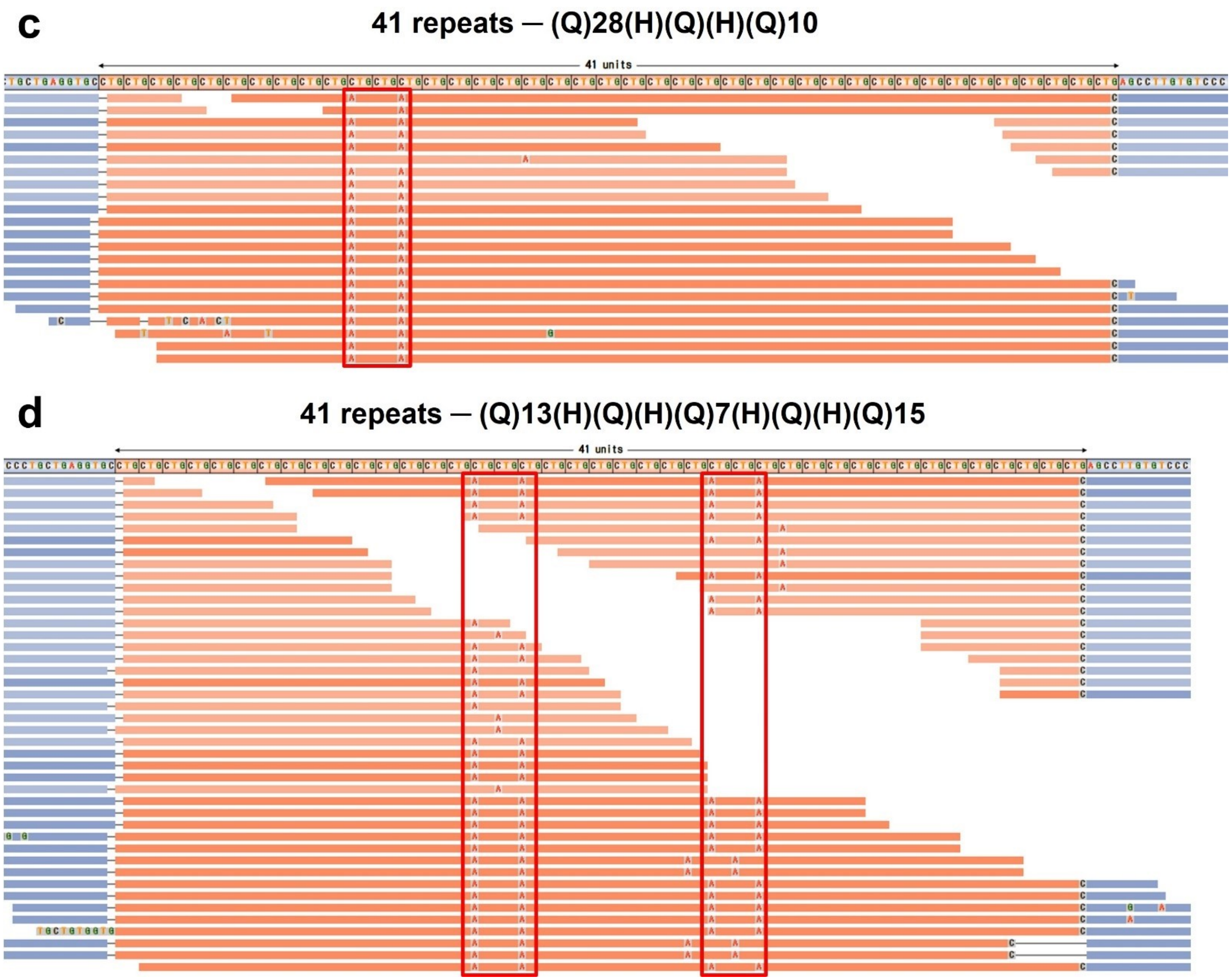
c
41 repeats — (Q)28(H)(Q)(H)(Q)10
41 units
d
41 repeats — (Q)13(H)(Q)(H)(Q)7(H)(Q)(H)(Q)15
41 units

**e** 39 repeats — (Q)26(H)(Q)(H)(Q)10

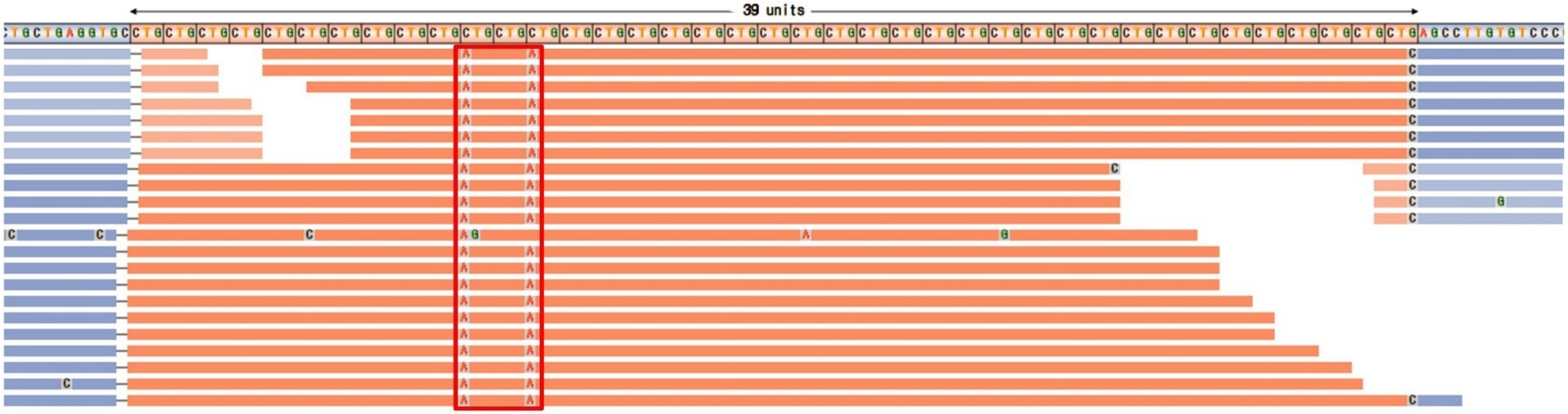


**f** 39 repeats — (Q)12(H)(Q)9(H)(Q)16

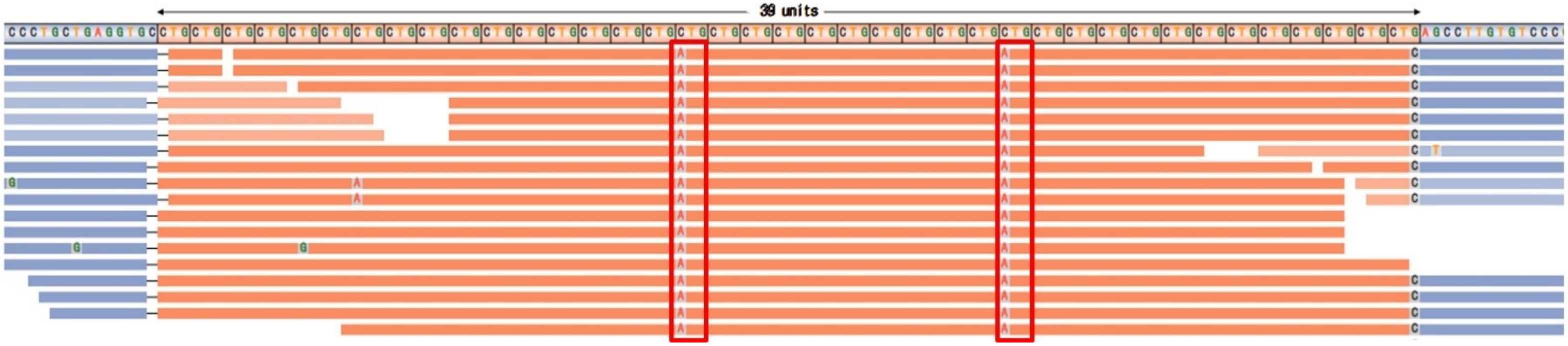

**Supplementary Table 1. Overview of the exome-sequenced cohort**

| Characteristics | *n* | % |
|---|---|---|
| **Probands** | | |
| Singleton (proband-only) | 709 | 28.2 |
| Duo (proband + single parent) | 55 | 2.2 |
| Trio (proband + both parents) | 1,704 | 67.9 |
| Quartet (trio + sibling) | 42 | 1.7 |
| **Total families** | 2,510 | 100.0 |
| **Sex** | | |
| Male | 1,379 | 54.9 |
| Female | 1,133 | 45.1 |
| **Onset-age** | | |
| Pediatric (<18 yrs) | 2,360 | 94.0 |
| Adult (≥18 yrs) | 150 | 6.0 |
| **Primary disease category** | | |
| Neurodevelopmental disorders | 1,647 | 65.6 |
| Neuromuscular diseases | 315 | 12.5 |
| Cerebellar ataxia | 82 | 3.3 |
| Hereditary spastic paraplegia | 56 | 2.2 |
| Others | 410 | 16.3 |
| **Total exomes** | 6,099 | |

## Supplementary Table 2. Targeted regions for short-tandem repeat analysis

| No. | Gene | Chr | Start | End | Disease (#OMIM) | MOI | Repeat unit | Location | Pathogenic threshold[a] |
|---|---|---|---|---|---|---|---|---|---|
| 1 | *AR* | chrX | 67545318 | 67545386 | SBMA (#313200) | XLR | CAG | Exon | 38 |
| 2 | *ARX* | chrX | 25013650 | 25013697 | EIEE1/XLID (#308350, #300419, #30021) | XL | GCC | Exon | 27 |
| 3 | *ATN1* | chr12 | 6936717 | 6936773 | DRPLA (#125370) | AD | CAG | Exon | 48 |
| 4 | *ATXN1* | chr6 | 16327636 | 16327725 | SCA1 (#164400) | AD | CAG | Exon | 39 |
| 5 | *ATXN3* | chr14 | 92071011 | 92071052 | SCA3 (#109150) | AD | CAG | Exon | 53 |
| 6 | *ATXN7* | chr3 | 63912686 | 63912715 | SCA7 (#164500) | AD | CAG | Exon | 34 |
| 7 | *CACNA1A* | chr19 | 13207858 | 13207897 | SCA6 (183086) | AD | CAG | Exon | 19 |
| 8 | *DMPK* | chr19 | 45770204 | 45770264 | DM1 (#160900) | AD | CTG | 3' UTR | 50 |
| 9 | *FOXL2* | chr3 | 138946022 | 138946063 | BPES (#110100) | AD/AR | GCG | Exon | 22 |
| 10 | *HTT_1* | chr4 | 3074877 | 3074939 | HD (#143100) | AD | CAG | Exon | 40 |
| 11 | *HTT_2* | chr4 | 3074940 | 3074966 | HD (#143100) | AD | CCG | Exon | 40 |
| 12 | *JPH3* | chr16 | 87604287 | 87604329 | HDL2 (#606438) | AD | CTG | Exon | 40 |
| 13 | *NOP56* | chr20 | 2652733 | 2652775 | SCA36 (#614153) | AD | GGCCTG | Intron 1 | 650 |
| 14 | *NOTCH2NLC* | chr1 | 149390803 | 149390842 | NIID (#603472) | AD | CGG | 5' UTR | 66 |
| 15 | *PHOX2B* | chr4 | 41745976 | 41746022 | CCHS (#209880) | AD | GCG | Exon | 24 |
| 16 | *PPP2R2B* | chr5 | 146878729 | 146878758 | SCA12 (#604326) | AD | CAG | 5' UTR | 51 |
| 17 | *PRDM12* | chr9 | 130681606 | 130681641 | HSAN8 (#616488) | AR | GCG | Exon | 18 |
| 18 | *SOX3* | chr3 | 181712418 | 181712456 | XLMR (#300123) | XLR | GCG | Exon | 22 |
| 19 | *TBP* | chr6 | 170561907 | 170562017 | SCA17 (#607136) | AD | CAG | Exon | 49 |
| 20 | *TCF4* | chr18 | 55222184 | 55635956 | FECD3 (#613267) | AD | TGC | Intron | 50 |
| 21 | *ZIC2* | chr13 | 99985449 | 99985494 | HPE5 (#609637) | AD | GCG | Exon | 25 |

This table is sourced from the previous report.[6] [a]The pathogenic ranges differ across studies, with the upper limit frequently unspecified. It is important to understand that these are only potentially pathogenic. Furthermore, alleles below these ranges can be associated with intermediate or premutation conditions.
Abbreviations: Online Mendelian Inheritance in Man, OMIM; mode of inheritance, MOI; autosomal dominant, AD; autosomal recessive, AR; X-linked, XL; X-linked recessive, XLR; untranslated region, UTR.

## Supplementary Table 3. Frequencies of *ATXN1* expanded alleles with CAT interruptions

| Repeat counts | Motifs | His residues | Observed allele counts | Individual (Family:Relationship) | Sex/Age[a] | Unrelated allele counts | Total unrelated individuals[b] | Allele frequency (%)[c] |
|---|---|---|---|---|---|---|---|---|
| 44 | $(Q)_{31}(H)(Q)(H)(Q)_{10}$ | 2 | 1 | F1069:Mother | F/40 | 1 | 4,256 | 0.0117 |
| 43 | $(Q)_{30}(H)(Q)(H)(Q)_{10}$ | 2 | 2 | F0654:Mother | F/44 | 1 | 4,256 | 0.0117 |
| | | | | F0654:Proband | F/14 | | | |
| 41 | $(Q)_{28}(H)(Q)(H)(Q)_{10}$ | 2 | 4 | F0050:Father | M/45 | 2 | 4,256 | 0.0235 |
| | | | | F0050:Proband | F/11 | | | |
| | | | | F1314:Mother | F/42 | | | |
| | | | | F1314:Proband | M/5 | | | |
| | $(Q)_{13}(H)(Q)(H)(Q)_{7}(H)(Q)(H)(Q)_{15}$ | 4 | 1 | F1372:Father | M/44 | 1 | 4,256 | 0.0117 |
| 39 | $(Q)_{26}(H)(Q)(H)(Q)_{10}$ | 2 | 2 | F0071:Mother | F/46 | 2 | 4,256 | 0.0235 |
| | | | | F1310:Father | M/50 | | | |
| | $(Q)_{12}(H)(Q)_{9}(H)(Q)_{16}$ | 2 | 2 | F0593:Mother | F/39 | 1 | 4,256 | 0.0117 |
| | | | | F0593:Proband | M/8 | | | |
| Total | - | - | 12 | | | 8 | 4,256 | 0.0939 |

[a]Age at evaluation. All individuals exhibited no clinical features associated with spinocerebellar ataxia type 1 (SCA1) until this age.
[b]Since fathers and mothers within a family were unrelated (no consanguineous marriages were observed in this cohort), the number of unrelated individuals could be one in singleton or duo-sequenced samples, and two in trio- or quartet-sequenced samples. Therefore, the total number of unrelated individuals was calculated as follows: {(No. of singleton or duo) + (No. of trio or quartet) x 2} = 4,256. See **Supplementary Table 1** for the corresponding numbers. [c]The allele frequency was calculated by dividing the count of unrelated alleles by the total count of unrelated alleles (the total number of unrelated individuals x 2).
